# Supplementary figures and images for: Drug-resistant tuberculosis care and treatment outcomes over the last 15 years in Ethiopia: Results from a mixed-method review of trends
Source: PLoS One. 2024 Aug 26;19(8):e0306076. doi: 10.1371/journal.pone.0306076 (PMC11346926; doi:10.1371/journal.pone.0306076)

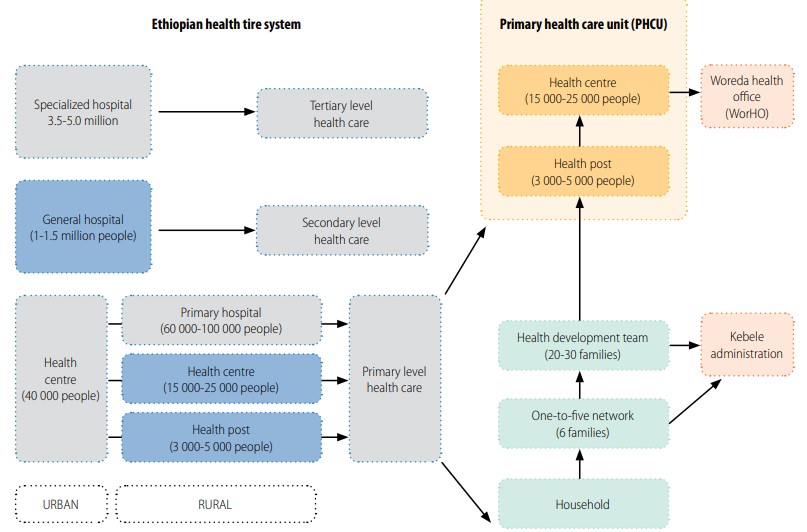

Supplement: S1 Fig — Available at file:///C:/Users/ddare/AppData/Local/Temp/MicrosoftEdgeDownloads/b3197175-c020-40b1-b0e3-0910f2ba0499/WHO-HIS-HSR-17.31-eng.pdf. (JPG) [file pone.0306076.s001.jpg]
